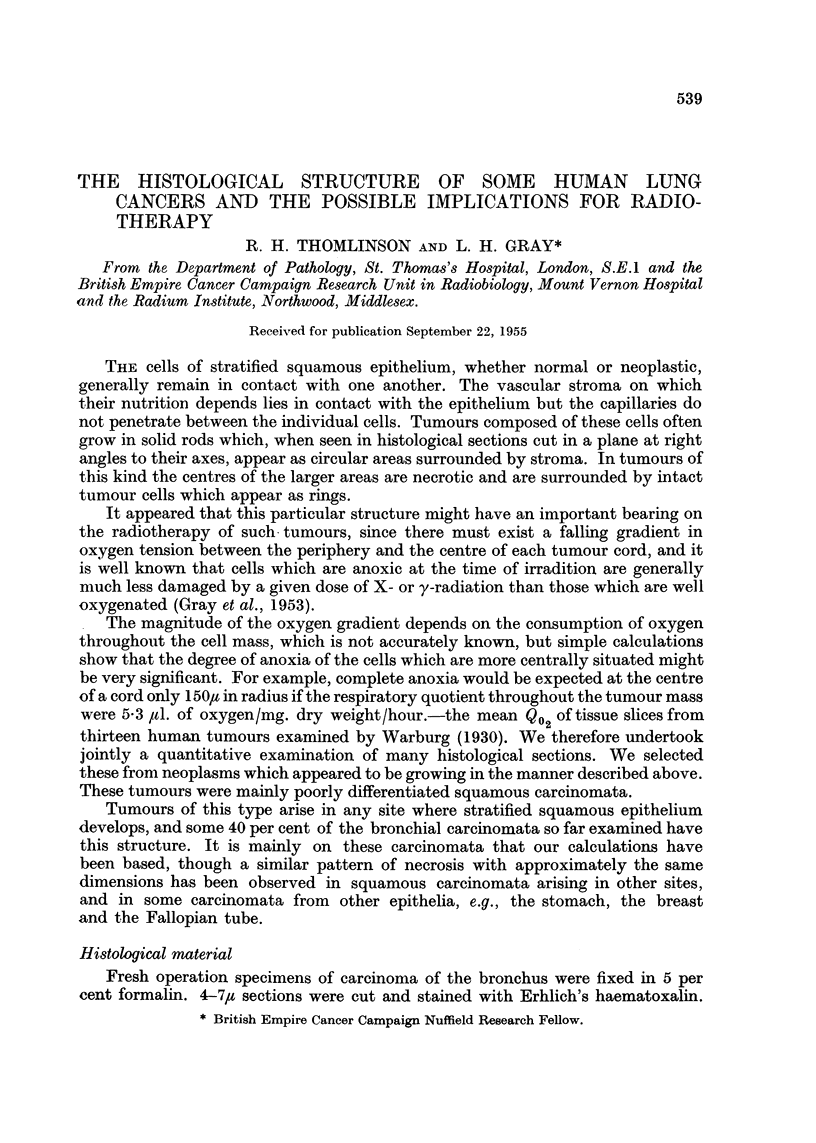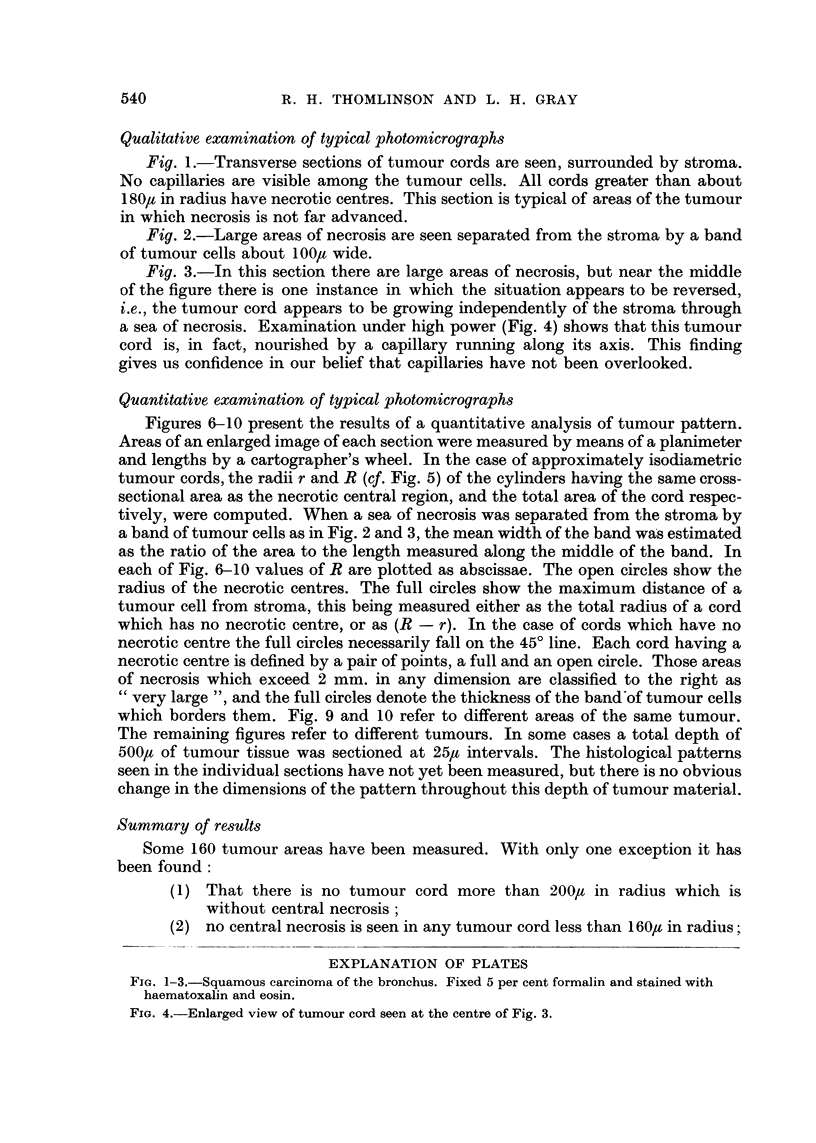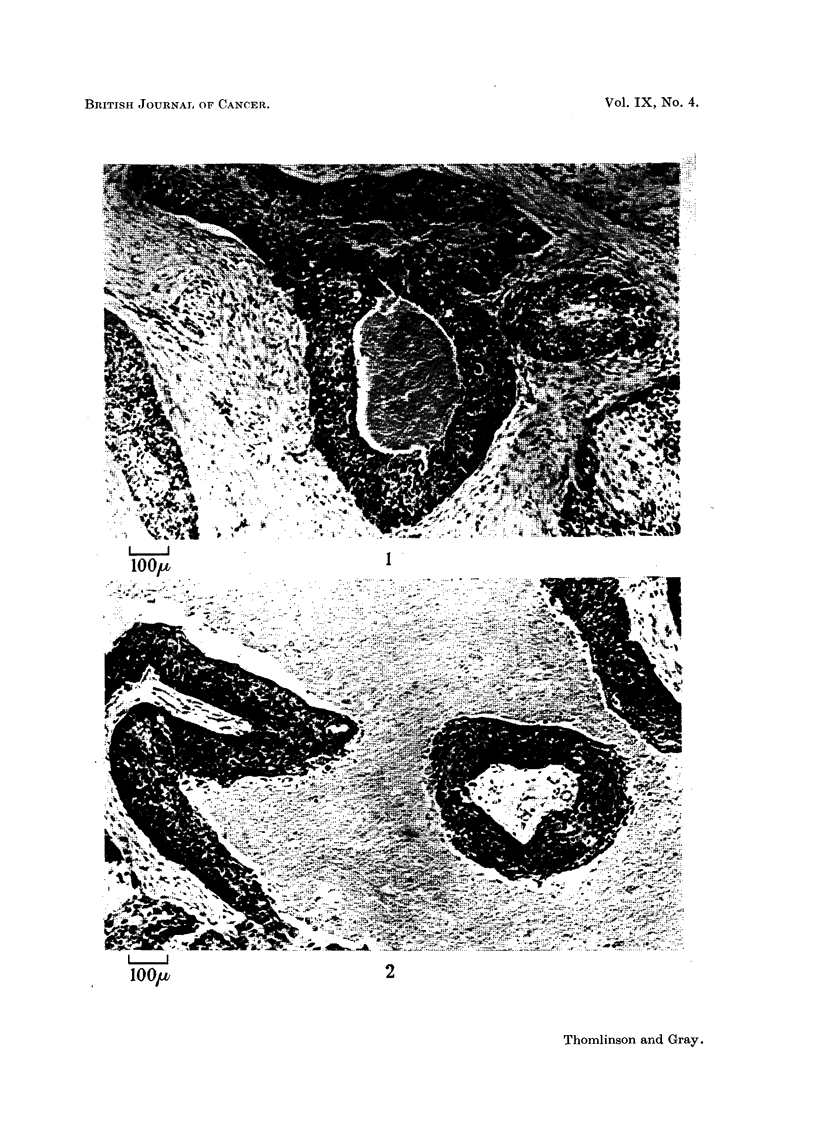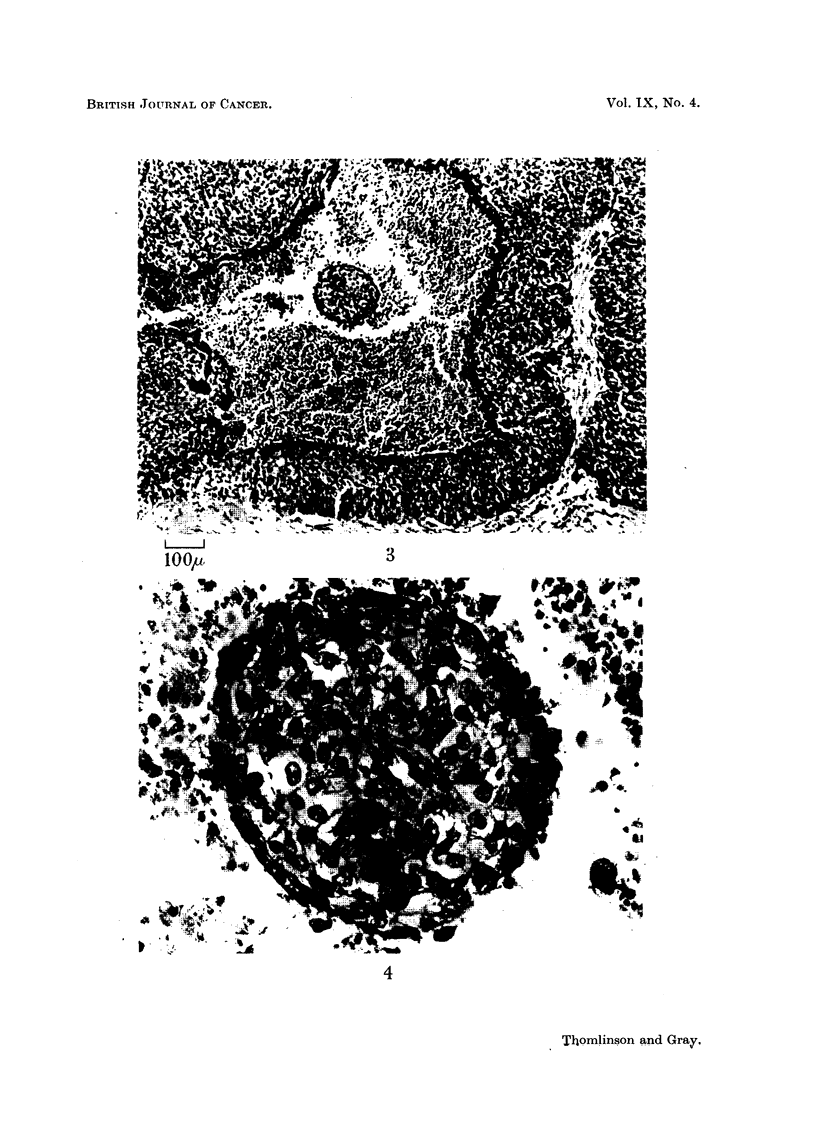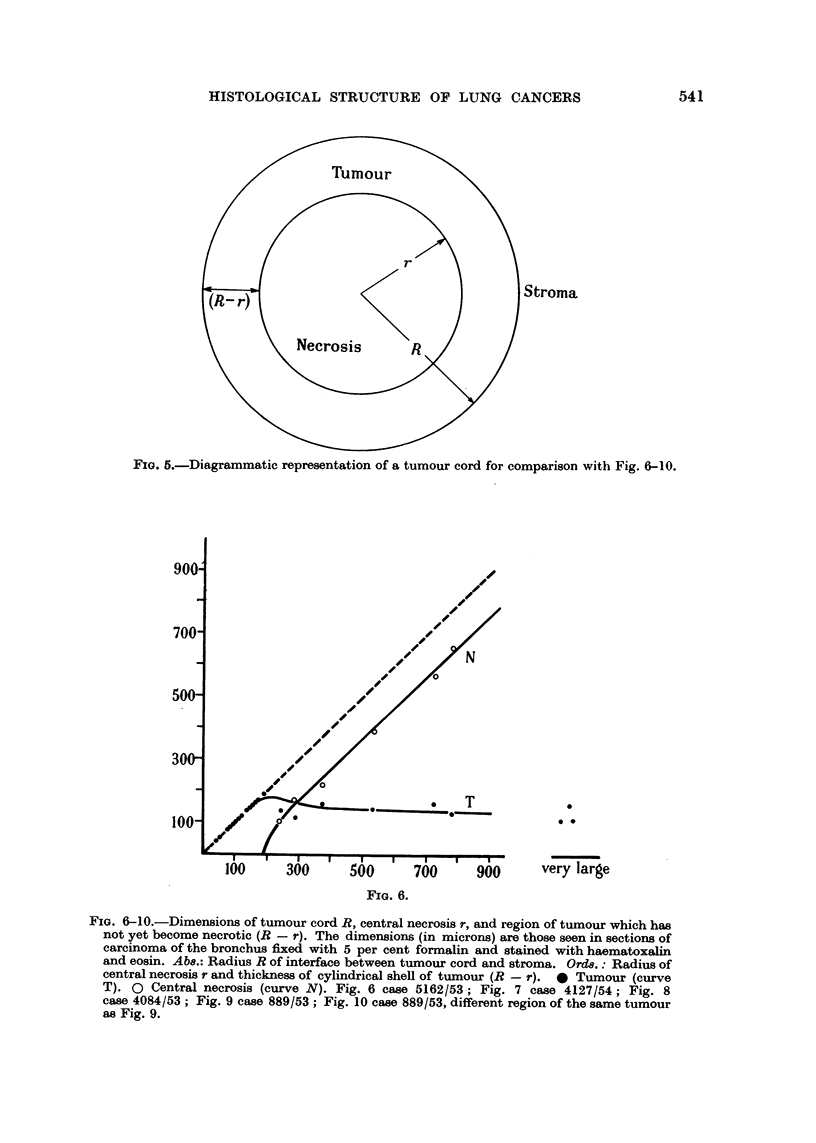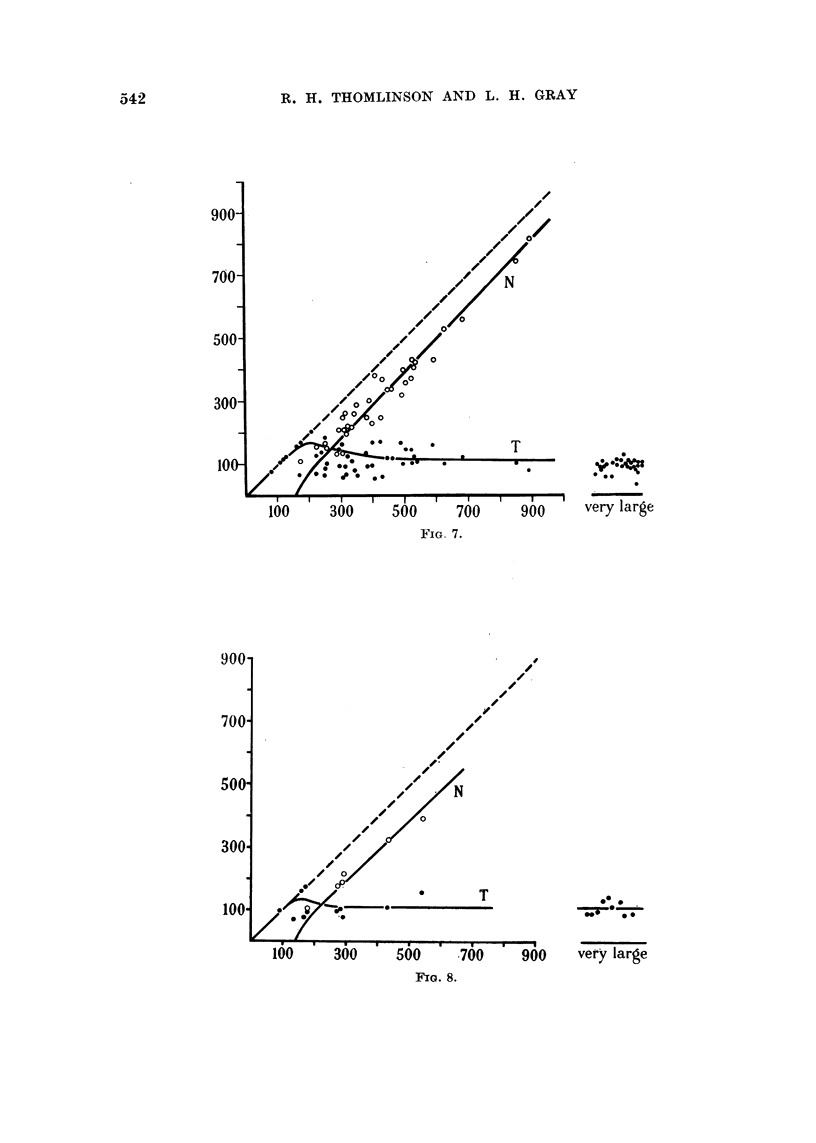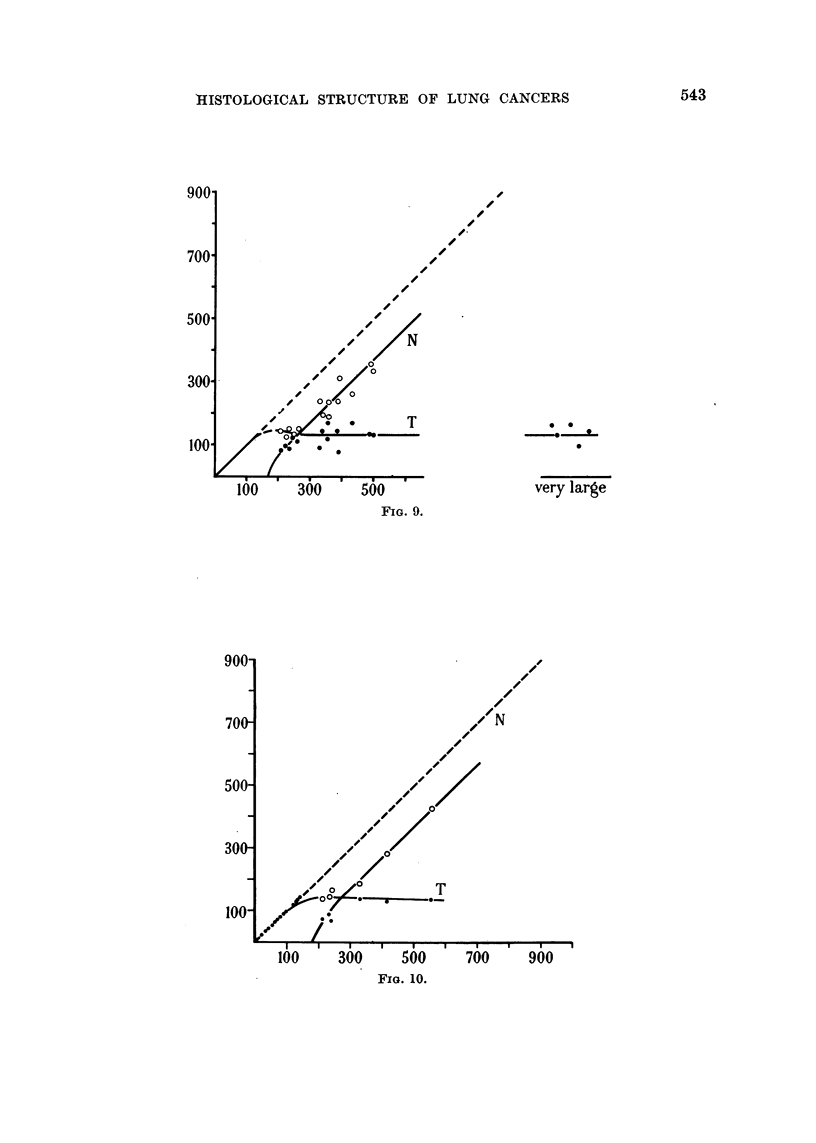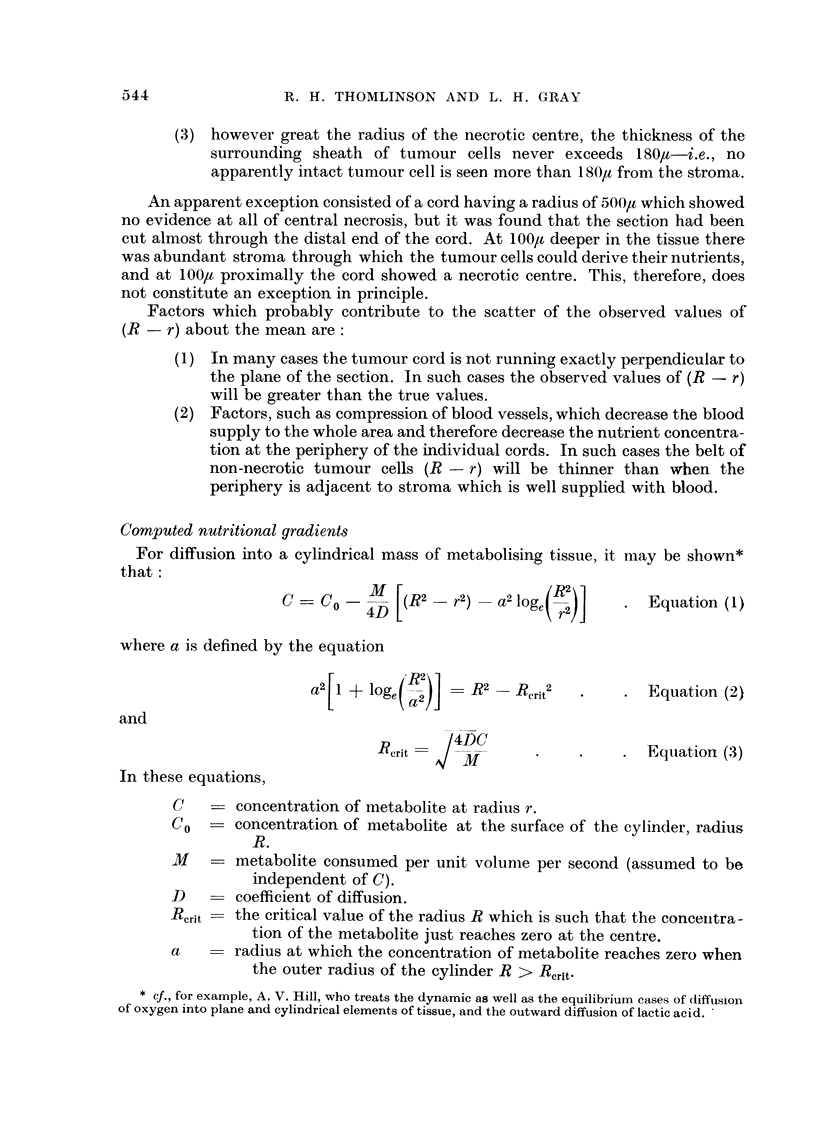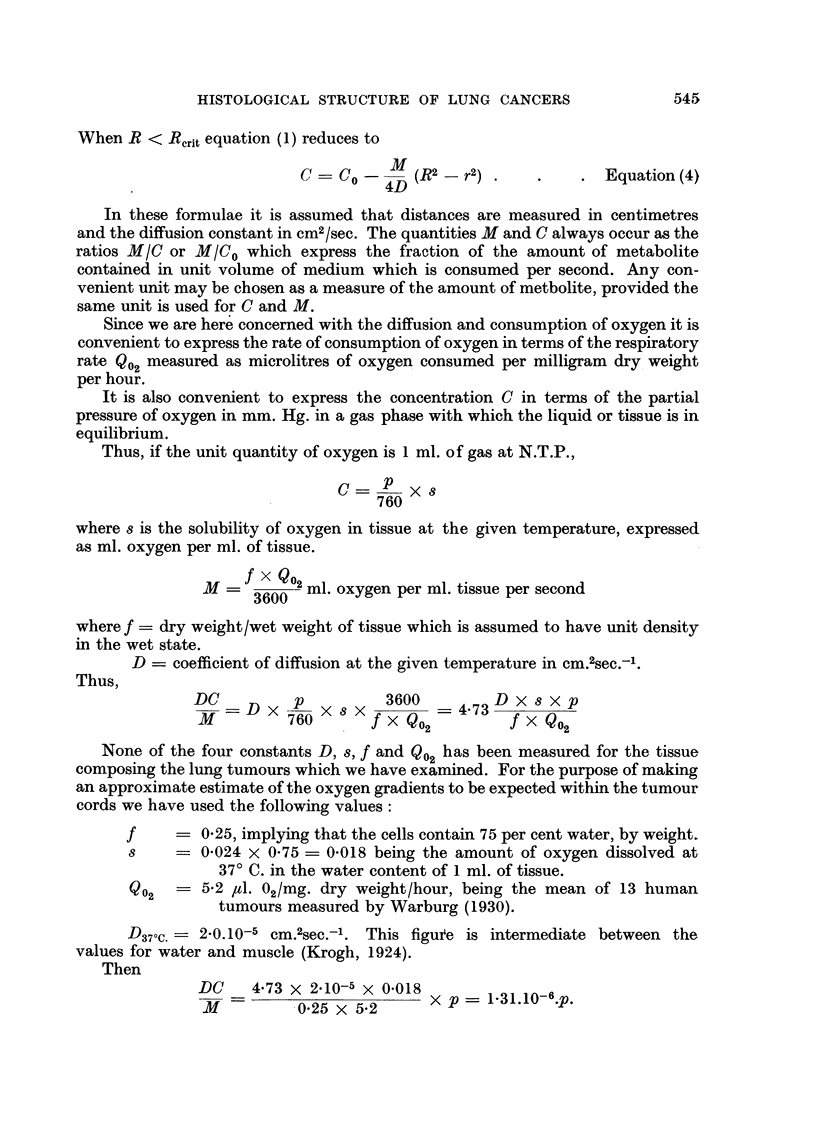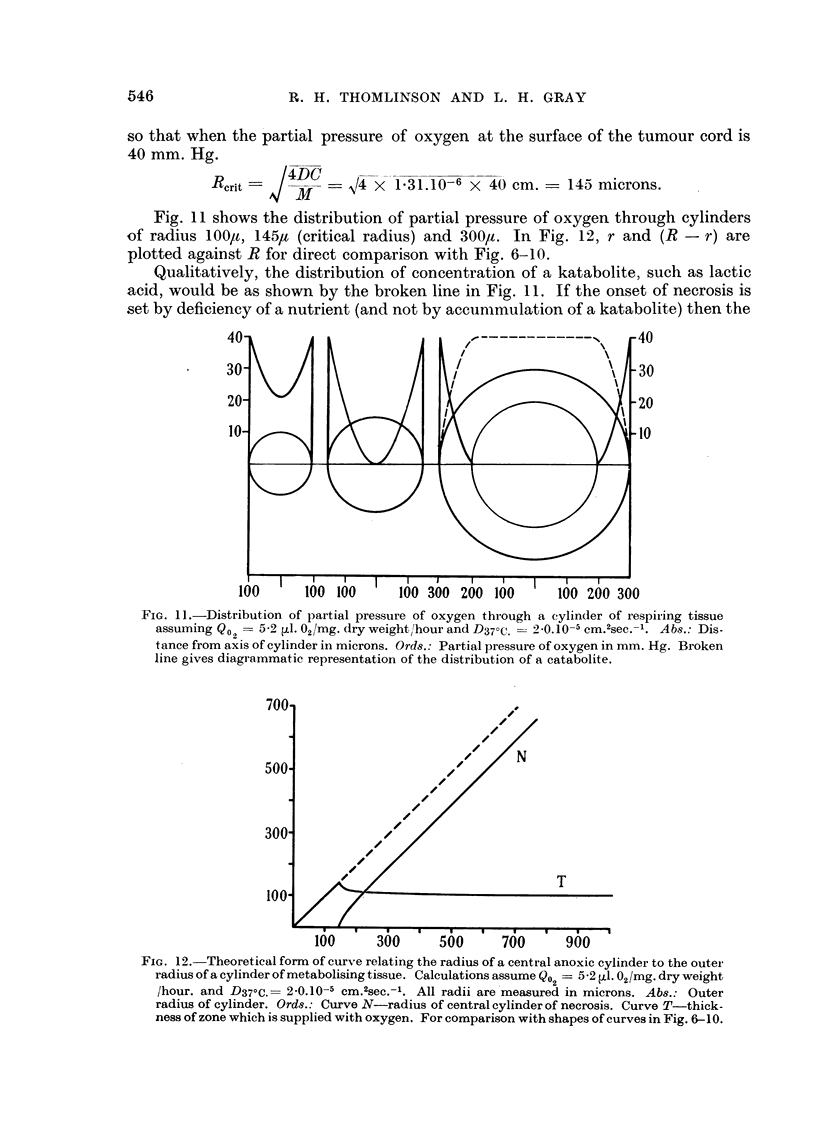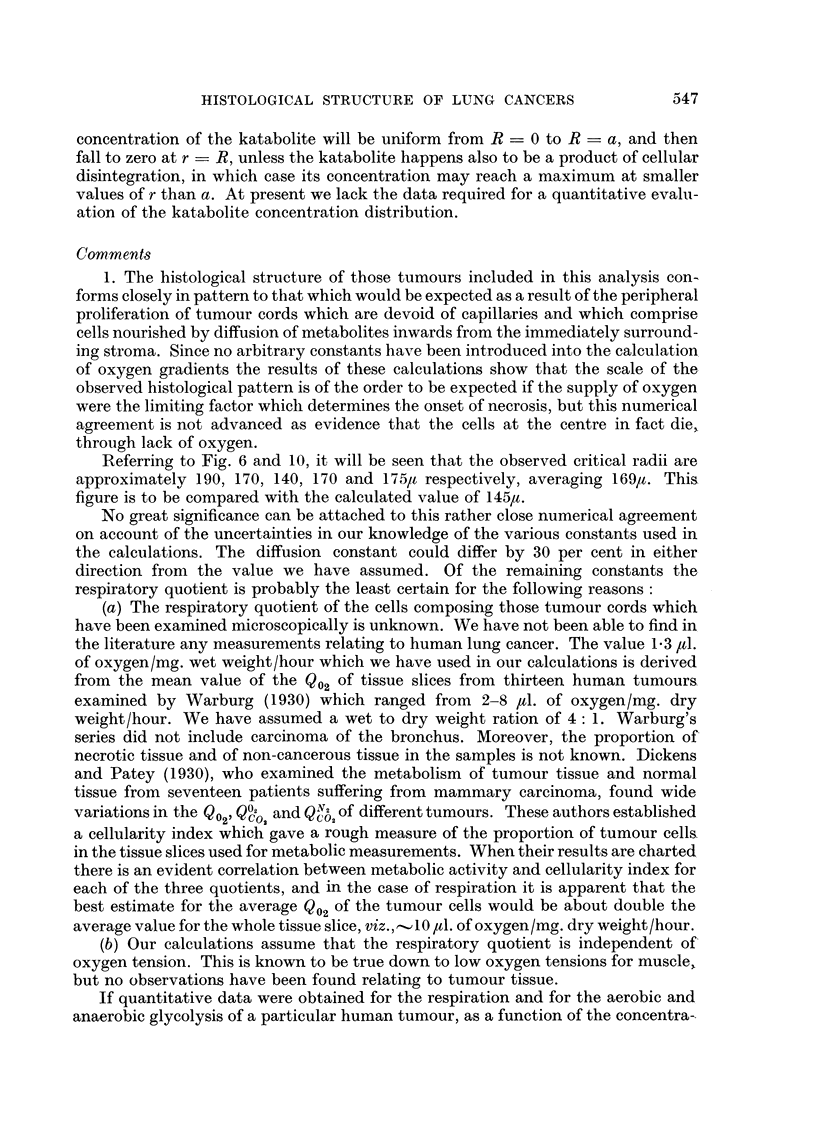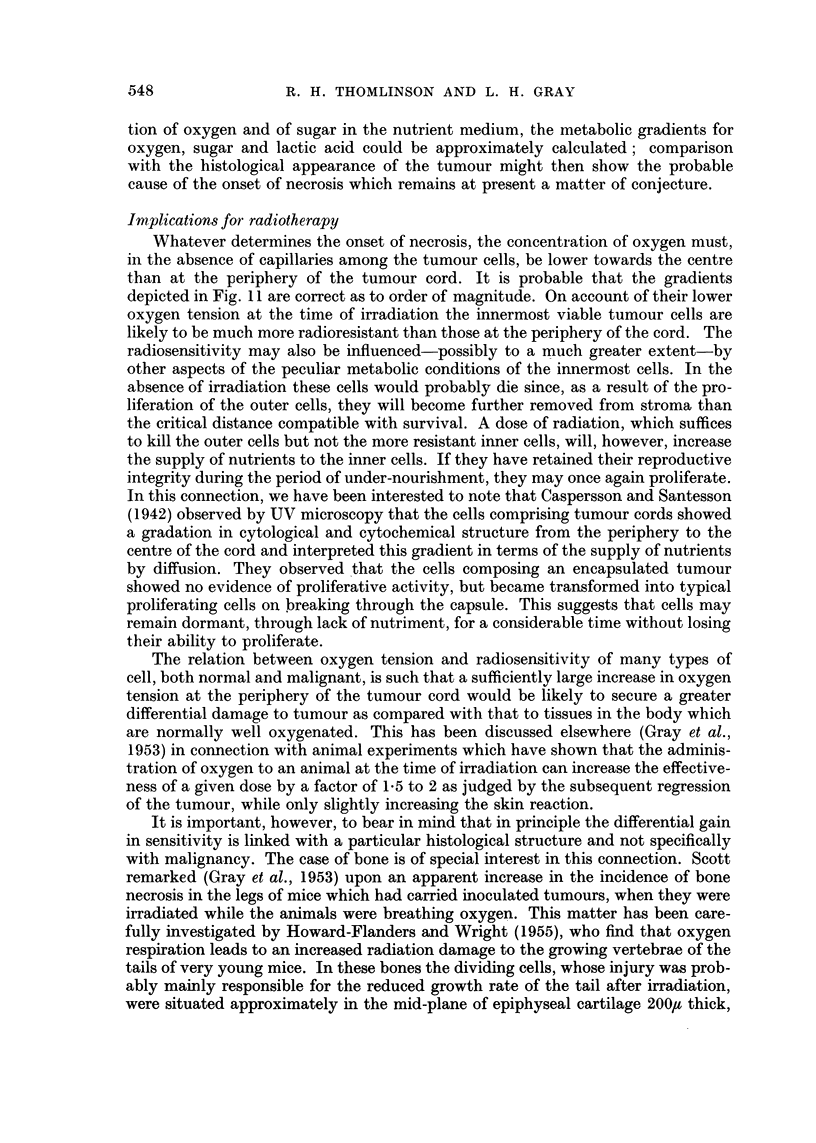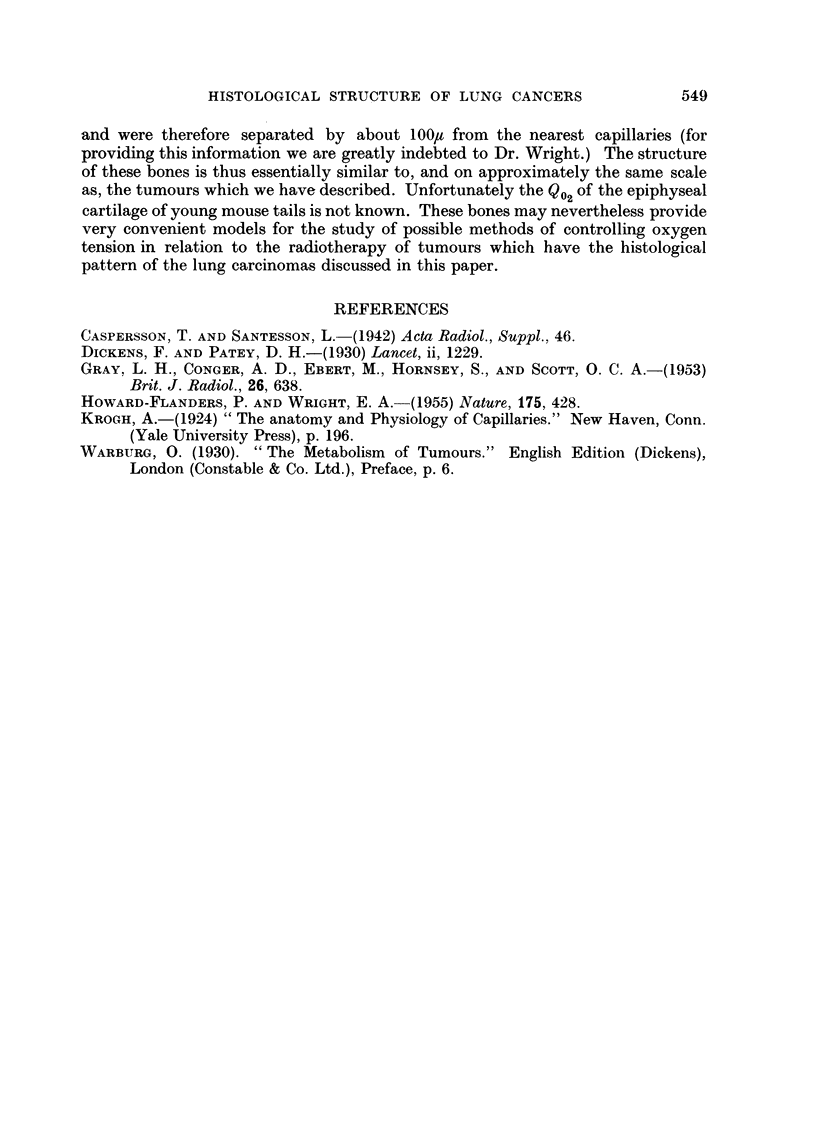# The Histological Structure of Some Human Lung Cancers and the Possible Implications for Radiotherapy

**DOI:** 10.1038/bjc.1955.55

**Published:** 1955-12

**Authors:** R. H. Thomlinson, L. H. Gray

## Abstract

**Images:**


### THE HISTOLOGICAL STRUCTURE OF SOME HUMAN LUNG CANCERS AND THE POSSIBLE IMPLICATIONS FOR RADIOTHERAPY

R. H. THOMLINSON AND L. H. GRAY*

*From the Department of Pathology, St. Thomas's Hospital, London, S.E.*1 *and the British Empire Cancer Campaign Research Unit in Radiobiology, Mount Vernon Hospital and the Radium Institute, Northwood, Middlesex.*

Received for publication September 22, 1955

THE cells of stratified squamous epithelium, whether normal or neoplastic, generally remain in contact with one another. The vascular stroma on which their nutrition depends lies in contact with the epithelium but the capillaries do not penetrate between the individual cells. Tumours composed of these cells often grow in solid rods which, when seen in histological sections cut in a plane at right angles to their axes, appear as circular areas surrounded by stroma. In tumours of this kind the centres of the larger areas are necrotic and are surrounded by intact tumour cells which appear as rings.

It appeared that this particular structure might have an important bearing on the radiotherapy of such tumours, since there must exist a falling gradient in oxygen tension between the periphery and the centre of each tumour cord, and it is well known that cells which are anoxic at the time of irradition are generally much less damaged by a given dose of X- or $\gamma$-radiation than those which are well oxygenated (Gray *et al.*, 1953).

The magnitude of the oxygen gradient depends on the consumption of oxygen throughout the cell mass, which is not accurately known, but simple calculations show that the degree of anoxia of the cells which are more centrally situated might be very significant. For example, complete anoxia would be expected at the centre of a cord only $150\mu$ in radius if the respiratory quotient throughout the tumour mass were 5·3 $\mu$l. of oxygen/mg. dry weight/hour.—the mean $Q_{0_2}$ of tissue slices from thirteen human tumours examined by Warburg (1930). We therefore undertook jointly a quantitative examination of many histological sections. We selected these from neoplasms which appeared to be growing in the manner described above. These tumours were mainly poorly differentiated squamous carcinomata.

Tumours of this type arise in any site where stratified squamous epithelium develops, and some 40 per cent of the bronchial carcinomata so far examined have this structure. It is mainly on these carcinomata that our calculations have been based, though a similar pattern of necrosis with approximately the same dimensions has been observed in squamous carcinomata arising in other sites, and in some carcinomata from other epithelia, *e.g.*, the stomach, the breast and the Fallopian tube.

### *Histological material*

Fresh operation specimens of carcinoma of the bronchus were fixed in 5 per cent formalin. 4–7$\mu$ sections were cut and stained with Erhlich's haematoxalin.

* British Empire Cancer Campaign Nuffield Research Fellow.

*Qualitative examination of typical photomicrographs*

*Fig.* 1.—Transverse sections of tumour cords are seen, surrounded by stroma. No capillaries are visible among the tumour cells. All cords greater than about 180$\mu$ in radius have necrotic centres. This section is typical of areas of the tumour in which necrosis is not far advanced.

*Fig.* 2.—Large areas of necrosis are seen separated from the stroma by a band of tumour cells about 100$\mu$ wide.

*Fig.* 3.—In this section there are large areas of necrosis, but near the middle of the figure there is one instance in which the situation appears to be reversed, *i.e.*, the tumour cord appears to be growing independently of the stroma through a sea of necrosis. Examination under high power (Fig. 4) shows that this tumour cord is, in fact, nourished by a capillary running along its axis. This finding gives us confidence in our belief that capillaries have not been overlooked.

*Quantitative examination of typical photomicrographs*

Figures 6–10 present the results of a quantitative analysis of tumour pattern. Areas of an enlarged image of each section were measured by means of a planimeter and lengths by a cartographer's wheel. In the case of approximately isodiametric tumour cords, the radii $r$ and $R$ (*cf.* Fig. 5) of the cylinders having the same cross-sectional area as the necrotic central region, and the total area of the cord respectively, were computed. When a sea of necrosis was separated from the stroma by a band of tumour cells as in Fig. 2 and 3, the mean width of the band was estimated as the ratio of the area to the length measured along the middle of the band. In each of Fig. 6–10 values of $R$ are plotted as abscissae. The open circles show the radius of the necrotic centres. The full circles show the maximum distance of a tumour cell from stroma, this being measured either as the total radius of a cord which has no necrotic centre, or as $(R - r)$. In the case of cords which have no necrotic centre the full circles necessarily fall on the 45° line. Each cord having a necrotic centre is defined by a pair of points, a full and an open circle. Those areas of necrosis which exceed 2 mm. in any dimension are classified to the right as " very large ", and the full circles denote the thickness of the band of tumour cells which borders them. Fig. 9 and 10 refer to different areas of the same tumour. The remaining figures refer to different tumours. In some cases a total depth of 500$\mu$ of tumour tissue was sectioned at 25$\mu$ intervals. The histological patterns seen in the individual sections have not yet been measured, but there is no obvious change in the dimensions of the pattern throughout this depth of tumour material.

*Summary of results*

Some 160 tumour areas have been measured. With only one exception it has been found :

(1) That there is no tumour cord more than 200$\mu$ in radius which is without central necrosis ;

(2) no central necrosis is seen in any tumour cord less than 160$\mu$ in radius;

---

EXPLANATION OF PLATES

Fig. 1–3.—Squamous carcinoma of the bronchus. Fixed 5 per cent formalin and stained with haematoxalin and eosin.

Fig. 4.—Enlarged view of tumour cord seen at the centre of Fig. 3.

100$\mu$ 1

100$\mu$ 2

Thomlinson and Gray.

3

4

FIG. 5.—Diagrammatic representation of a tumour cord for comparison with Fig. 6–10.

FIG. 6.

FIG. 6–10.—Dimensions of tumour cord $R$, central necrosis $r$, and region of tumour which has not yet become necrotic ($R - r$). The dimensions (in microns) are those seen in sections of carcinoma of the bronchus fixed with 5 per cent formalin and stained with haematoxalin and eosin. *Abs.*: Radius $R$ of interface between tumour cord and stroma. *Ords.*: Radius of central necrosis $r$ and thickness of cylindrical shell of tumour ($R - r$). ● Tumour (curve T). ○ Central necrosis (curve $N$). Fig. 6 case 5162/53 ; Fig. 7 case 4127/54 ; Fig. 8 case 4084/53 ; Fig. 9 case 889/53 ; Fig. 10 case 889/53, different region of the same tumour as Fig. 9.

900

700

500

300

100

N

T

100

300

500

700

900

very large

FIG. 7.

900

700

500

300

100

N

T

100

300

500

700

900

very large

FIG. 8.

900

700

500

300

100

100

300

500

N

T

very large

FIG. 9.

900

700

500

300

100

100

300

500

700

900

N

T

FIG. 10.

(3) however great the radius of the necrotic centre, the thickness of the surrounding sheath of tumour cells never exceeds $180\mu$—*i.e.*, no apparently intact tumour cell is seen more than $180\mu$ from the stroma.

An apparent exception consisted of a cord having a radius of $500\mu$ which showed no evidence at all of central necrosis, but it was found that the section had been cut almost through the distal end of the cord. At $100\mu$ deeper in the tissue there was abundant stroma through which the tumour cells could derive their nutrients, and at $100\mu$ proximally the cord showed a necrotic centre. This, therefore, does not constitute an exception in principle.

Factors which probably contribute to the scatter of the observed values of $(R - r)$ about the mean are :

(1) In many cases the tumour cord is not running exactly perpendicular to the plane of the section. In such cases the observed values of $(R - r)$ will be greater than the true values.

(2) Factors, such as compression of blood vessels, which decrease the blood supply to the whole area and therefore decrease the nutrient concentration at the periphery of the individual cords. In such cases the belt of non-necrotic tumour cells $(R - r)$ will be thinner than when the periphery is adjacent to stroma which is well supplied with blood.

*Computed nutritional gradients*

For diffusion into a cylindrical mass of metabolising tissue, it may be shown* that :

$$C = C_0 - \frac{M}{4D}\left[(R^2 - r^2) - a^2 \log_e\left(\frac{R^2}{r^2}\right)\right] \quad . \quad \text{Equation (1)}$$

where $a$ is defined by the equation

$$a^2\left[1 + \log_e\left(\frac{R^2}{a^2}\right)\right] = R^2 - R_{\text{crit}}^2 \quad . \quad . \quad \text{Equation (2)}$$

and

$$R_{\text{crit}} = \sqrt{\frac{4DC}{M}} \quad . \quad . \quad . \quad \text{Equation (3)}$$

In these equations,

$C$ = concentration of metabolite at radius $r$.
$C_0$ = concentration of metabolite at the surface of the cylinder, radius $R$.
$M$ = metabolite consumed per unit volume per second (assumed to be independent of $C$).
$D$ = coefficient of diffusion.
$R_{\text{crit}}$ = the critical value of the radius $R$ which is such that the concentration of the metabolite just reaches zero at the centre.
$a$ = radius at which the concentration of metabolite reaches zero when the outer radius of the cylinder $R > R_{\text{crit}}$.

* *cf.*, for example, A. V. Hill, who treats the dynamic as well as the equilibrium cases of diffusion of oxygen into plane and cylindrical elements of tissue, and the outward diffusion of lactic acid.

When $R < R_{\text{crit}}$ equation (1) reduces to

$$C = C_0 - \frac{M}{4D}(R^2 - r^2) \quad . \quad . \quad . \quad \text{Equation (4)}$$

In these formulae it is assumed that distances are measured in centimetres and the diffusion constant in $\text{cm}^2/\text{sec}$. The quantities $M$ and $C$ always occur as the ratios $M/C$ or $M/C_0$ which express the fraction of the amount of metabolite contained in unit volume of medium which is consumed per second. Any convenient unit may be chosen as a measure of the amount of metbolite, provided the same unit is used for $C$ and $M$.

Since we are here concerned with the diffusion and consumption of oxygen it is convenient to express the rate of consumption of oxygen in terms of the respiratory rate $Q_{0_2}$ measured as microlitres of oxygen consumed per milligram dry weight per hour.

It is also convenient to express the concentration $C$ in terms of the partial pressure of oxygen in mm. Hg. in a gas phase with which the liquid or tissue is in equilibrium.

Thus, if the unit quantity of oxygen is 1 ml. of gas at N.T.P.,

$$C = \frac{p}{760} \times s$$

where $s$ is the solubility of oxygen in tissue at the given temperature, expressed as ml. oxygen per ml. of tissue.

$$M = \frac{f \times Q_{0_2}}{3600} \text{ ml. oxygen per ml. tissue per second}$$

where $f$ = dry weight/wet weight of tissue which is assumed to have unit density in the wet state.

$D$ = coefficient of diffusion at the given temperature in $\text{cm.}^2\text{sec.}^{-1}$.

Thus,

$$\frac{DC}{M} = D \times \frac{p}{760} \times s \times \frac{3600}{f \times Q_{0_2}} = 4{\cdot}73 \frac{D \times s \times p}{f \times Q_{0_2}}$$

None of the four constants $D$, $s$, $f$ and $Q_{0_2}$ has been measured for the tissue composing the lung tumours which we have examined. For the purpose of making an approximate estimate of the oxygen gradients to be expected within the tumour cords we have used the following values :

$f$ = 0·25, implying that the cells contain 75 per cent water, by weight.

$s$ = 0·024 × 0·75 = 0·018 being the amount of oxygen dissolved at 37° C. in the water content of 1 ml. of tissue.

$Q_{0_2}$ = 5·2 $\mu$l. $0_2$/mg. dry weight/hour, being the mean of 13 human tumours measured by Warburg (1930).

$D_{37^\circ\text{C.}}$ = $2{\cdot}0.10^{-5}$ $\text{cm.}^2\text{sec.}^{-1}$. This figure is intermediate between the values for water and muscle (Krogh, 1924).

Then

$$\frac{DC}{M} = \frac{4{\cdot}73 \times 2{\cdot}10^{-5} \times 0{\cdot}018}{0{\cdot}25 \times 5{\cdot}2} \times p = 1{\cdot}31.10^{-6}.p.$$

so that when the partial pressure of oxygen at the surface of the tumour cord is 40 mm. Hg.

$$R_{\text{crit}} = \sqrt{\frac{4DC}{M}} = \sqrt{4 \times 1{\cdot}31.10^{-6} \times 40} \text{ cm.} = 145 \text{ microns.}$$

Fig. 11 shows the distribution of partial pressure of oxygen through cylinders of radius $100\mu$, $145\mu$ (critical radius) and $300\mu$. In Fig. 12, $r$ and $(R - r)$ are plotted against $R$ for direct comparison with Fig. 6–10.

Qualitatively, the distribution of concentration of a katabolite, such as lactic acid, would be as shown by the broken line in Fig. 11. If the onset of necrosis is set by deficiency of a nutrient (and not by accummulation of a katabolite) then the

Fig. 11.—Distribution of partial pressure of oxygen through a cylinder of respiring tissue assuming $Q_{0_2} = 5{\cdot}2$ µl. $0_2$/mg. dry weight/hour and $D_{37°C.} = 2{\cdot}0.10^{-5}$ cm.²sec.⁻¹. *Abs.:* Distance from axis of cylinder in microns. *Ords.:* Partial pressure of oxygen in mm. Hg. Broken line gives diagrammatic representation of the distribution of a catabolite.

Fig. 12.—Theoretical form of curve relating the radius of a central anoxic cylinder to the outer radius of a cylinder of metabolising tissue. Calculations assume $Q_{0_2} = 5{\cdot}2$ µl. $0_2$/mg. dry weight /hour. and $D_{37°C.} = 2{\cdot}0.10^{-5}$ cm.²sec.⁻¹. All radii are measured in microns. *Abs.:* Outer radius of cylinder. *Ords.:* Curve *N*—radius of central cylinder of necrosis. Curve *T*—thickness of zone which is supplied with oxygen. For comparison with shapes of curves in Fig. 6–10.

concentration of the katabolite will be uniform from $R = 0$ to $R = a$, and then fall to zero at $r = R$, unless the katabolite happens also to be a product of cellular disintegration, in which case its concentration may reach a maximum at smaller values of $r$ than $a$. At present we lack the data required for a quantitative evaluation of the katabolite concentration distribution.

*Comments*

1. The histological structure of those tumours included in this analysis conforms closely in pattern to that which would be expected as a result of the peripheral proliferation of tumour cords which are devoid of capillaries and which comprise cells nourished by diffusion of metabolites inwards from the immediately surrounding stroma. Since no arbitrary constants have been introduced into the calculation of oxygen gradients the results of these calculations show that the scale of the observed histological pattern is of the order to be expected if the supply of oxygen were the limiting factor which determines the onset of necrosis, but this numerical agreement is not advanced as evidence that the cells at the centre in fact die through lack of oxygen.

Referring to Fig. 6 and 10, it will be seen that the observed critical radii are approximately 190, 170, 140, 170 and 175$\mu$ respectively, averaging 169$\mu$. This figure is to be compared with the calculated value of 145$\mu$.

No great significance can be attached to this rather close numerical agreement on account of the uncertainties in our knowledge of the various constants used in the calculations. The diffusion constant could differ by 30 per cent in either direction from the value we have assumed. Of the remaining constants the respiratory quotient is probably the least certain for the following reasons :

(*a*) The respiratory quotient of the cells composing those tumour cords which have been examined microscopically is unknown. We have not been able to find in the literature any measurements relating to human lung cancer. The value 1·3 $\mu$l. of oxygen/mg. wet weight/hour which we have used in our calculations is derived from the mean value of the $Q_{O_2}$ of tissue slices from thirteen human tumours examined by Warburg (1930) which ranged from 2–8 $\mu$l. of oxygen/mg. dry weight/hour. We have assumed a wet to dry weight ration of 4 : 1. Warburg's series did not include carcinoma of the bronchus. Moreover, the proportion of necrotic tissue and of non-cancerous tissue in the samples is not known. Dickens and Patey (1930), who examined the metabolism of tumour tissue and normal tissue from seventeen patients suffering from mammary carcinoma, found wide variations in the $Q_{O_2}$, $Q^{O_2}_{CO_2}$ and $Q^{N_2}_{CO_2}$ of different tumours. These authors established a cellularity index which gave a rough measure of the proportion of tumour cells in the tissue slices used for metabolic measurements. When their results are charted there is an evident correlation between metabolic activity and cellularity index for each of the three quotients, and in the case of respiration it is apparent that the best estimate for the average $Q_{O_2}$ of the tumour cells would be about double the average value for the whole tissue slice, *viz.*, $\sim$10 $\mu$l. of oxygen/mg. dry weight/hour.

(*b*) Our calculations assume that the respiratory quotient is independent of oxygen tension. This is known to be true down to low oxygen tensions for muscle, but no observations have been found relating to tumour tissue.

If quantitative data were obtained for the respiration and for the aerobic and anaerobic glycolysis of a particular human tumour, as a function of the concentration of oxygen and of sugar in the nutrient medium, the metabolic gradients for oxygen, sugar and lactic acid could be approximately calculated; comparison with the histological appearance of the tumour might then show the probable cause of the onset of necrosis which remains at present a matter of conjecture.

*Implications for radiotherapy*

Whatever determines the onset of necrosis, the concentration of oxygen must, in the absence of capillaries among the tumour cells, be lower towards the centre than at the periphery of the tumour cord. It is probable that the gradients depicted in Fig. 11 are correct as to order of magnitude. On account of their lower oxygen tension at the time of irradiation the innermost viable tumour cells are likely to be much more radioresistant than those at the periphery of the cord. The radiosensitivity may also be influenced—possibly to a much greater extent—by other aspects of the peculiar metabolic conditions of the innermost cells. In the absence of irradiation these cells would probably die since, as a result of the proliferation of the outer cells, they will become further removed from stroma than the critical distance compatible with survival. A dose of radiation, which suffices to kill the outer cells but not the more resistant inner cells, will, however, increase the supply of nutrients to the inner cells. If they have retained their reproductive integrity during the period of under-nourishment, they may once again proliferate. In this connection, we have been interested to note that Caspersson and Santesson (1942) observed by UV microscopy that the cells comprising tumour cords showed a gradation in cytological and cytochemical structure from the periphery to the centre of the cord and interpreted this gradient in terms of the supply of nutrients by diffusion. They observed that the cells composing an encapsulated tumour showed no evidence of proliferative activity, but became transformed into typical proliferating cells on breaking through the capsule. This suggests that cells may remain dormant, through lack of nutriment, for a considerable time without losing their ability to proliferate.

The relation between oxygen tension and radiosensitivity of many types of cell, both normal and malignant, is such that a sufficiently large increase in oxygen tension at the periphery of the tumour cord would be likely to secure a greater differential damage to tumour as compared with that to tissues in the body which are normally well oxygenated. This has been discussed elsewhere (Gray *et al.*, 1953) in connection with animal experiments which have shown that the administration of oxygen to an animal at the time of irradiation can increase the effectiveness of a given dose by a factor of 1·5 to 2 as judged by the subsequent regression of the tumour, while only slightly increasing the skin reaction.

It is important, however, to bear in mind that in principle the differential gain in sensitivity is linked with a particular histological structure and not specifically with malignancy. The case of bone is of special interest in this connection. Scott remarked (Gray *et al.*, 1953) upon an apparent increase in the incidence of bone necrosis in the legs of mice which had carried inoculated tumours, when they were irradiated while the animals were breathing oxygen. This matter has been carefully investigated by Howard-Flanders and Wright (1955), who find that oxygen respiration leads to an increased radiation damage to the growing vertebrae of the tails of very young mice. In these bones the dividing cells, whose injury was probably mainly responsible for the reduced growth rate of the tail after irradiation, were situated approximately in the mid-plane of epiphyseal cartilage $200\mu$ thick, and were therefore separated by about 100$\mu$ from the nearest capillaries (for providing this information we are greatly indebted to Dr. Wright.) The structure of these bones is thus essentially similar to, and on approximately the same scale as, the tumours which we have described. Unfortunately the $Q_{O_2}$ of the epiphyseal cartilage of young mouse tails is not known. These bones may nevertheless provide very convenient models for the study of possible methods of controlling oxygen tension in relation to the radiotherapy of tumours which have the histological pattern of the lung carcinomas discussed in this paper.

### REFERENCES

Caspersson, T. and Santesson, L.—(1942) *Acta Radiol., Suppl.*, 46.
Dickens, F. and Patey, D. H.—(1930) *Lancet*, ii, 1229.
Gray, L. H., Conger, A. D., Ebert, M., Hornsey, S., and Scott, O. C. A.—(1953) *Brit. J. Radiol.*, **26**, 638.
Howard-Flanders, P. and Wright, E. A.—(1955) *Nature*, **175**, 428.
Krogh, A.—(1924) " The anatomy and Physiology of Capillaries." New Haven, Conn. (Yale University Press), p. 196.
Warburg, O. (1930). " The Metabolism of Tumours." English Edition (Dickens), London (Constable & Co. Ltd.), Preface, p. 6.